# Magnetic Resonance Imaging of Electrolysis.

**DOI:** 10.1038/srep08095

**Published:** 2015-02-09

**Authors:** Arie Meir, Mohammad Hjouj, Liel Rubinsky, Boris Rubinsky

**Affiliations:** 1 Graduate Program in Biophysics, University of California Berkeley, Berkeley, CA 94720; 2 Medical Imaging Department; Faculty of Health Professions, Al-Quds University/Abu Dies/Jerusalem; 3 Department of Mechanical Engineering, University of California Berkeley, Berkeley, CA 94720

## Abstract

This study explores the hypothesis that Magnetic Resonance Imaging (MRI) can image the process of electrolysis by detecting pH fronts. The study has relevance to real time control of cell ablation with electrolysis. To investigate the hypothesis we compare the following MR imaging sequences: T1 weighted, T2 weighted and Proton Density (PD), with optical images acquired using pH-sensitive dyes embedded in a physiological saline agar solution phantom treated with electrolysis and discrete measurements with a pH microprobe. We further demonstrate the biological relevance of our work using a bacterial *E. Coli* model, grown on the phantom. The results demonstrate the ability of MRI to image electrolysis produced pH changes in a physiological saline phantom and show that these changes correlate with cell death in the *E. Coli* model grown on the phantom. The results are promising and invite further experimental research.

In a biomedical context, electrolysis refers to the passage of low magnitude, direct current between two electrodes through a living tissue. Electrolytic ablation is the application of electrolysis for inducing irreversible damage to undesired tissues, and research into the biochemical/biophysical aspects of tissue electrolysis can be traced to the 19^th^ century^1^. In modern medicine, the tools available for tissue ablation are based on various biophysical and biochemical processes. Thermally based approaches include heating, cooling or freezing, while electroporation, injection of chemical agents, sonoporation and photodynamic effects are additional examples for more recent developments which were suggested as ways to expand the surgical arsenal. Electrolysis is a part of this toolkit and the basic underlying mechanism revolves around the ionic species in the tissue, species, which change into compounds that can ablate cells.

A large body of the modern electrolysis research can be traced back to the work of Nordenstrom and colleagues^2^,^3^. To understand the effects of electrolysis on tissue, researchers apply tools like histology and mathematical models of the involved electrochemical processes^4^,^5^,^6^,^7^,^8^,^9^,^10^,^11^,^12^,^13^,^14^. Accompanying the more fundamental research efforts, there is a growing body of clinical work, e.g.^15^,^16^,^17^,^18^. This work was inspired by several findings and the development of a few research techniques: it was shown that the electrolysis induced pH changes can be used to reliably monitor the extent of tissue ablation^19^. These findings have led to several basic studies on quantifying the process of electrolysis through the use of transparent gels with pH dyes^11^,^20^,^21^. Expanding the view beyond electrolysis-driven pH changes, different approaches have been developed to allow monitoring of pH levels. These include classical instruments such as the glass electrode^22^, electrochemically based tools such as cyclic voltammetry^23^,^24^ or semiconductor based micro-electrode pH sensors^25^. These discrete pH-measuring devices typically have very good pH resolution. However, they generate discrete data and cannot provide a continuous, spatial image of the pH distribution. The devices are often sensitive, require recalibration and their response time, even in stirred solutions, is between 5 and 15 seconds. Furthermore, even though the probe tips can be of microscopic size (on the order of microns), the outer diameter of the device's body is on the order of a centimeters, which limits the spatial resolution. To summarize, pH meters are excellent devices for measuring local pH, when the pH does not change in time. However, in electrolytic ablation the continuous generation of electrolytic products and their diffusion over time through the treated volume leads to changes in local pH in both time and space. This requires developing a pH detection system that can provide a continuous temporal and spatial distribution of the pH changes. Therefore, while pH-measuring devices can generate excellent discrete local data, they cannot be used for continuous measurements in time (because of the long response time, tissue fouling issues and the need to recalibrate) or in space (because of their discrete nature and size) of the process of electrolysis. The goal of this study is to introduce a technology than can detect changes in pH in both time and space, to be used as a tool in the context of electrolytic treatment of tissues.

Medical imaging has been one of the key innovation drivers in the minimally invasive surgery field. Minimally invasive procedures are now part of a common practice for ablating tissue deep inside the body, but most of these procedures cannot be performed without medical imaging^26^. Electrolysis is currently limited by the lack of an effective means to monitor the extent of tissue ablation deep in the body. During electrolysis, pH fronts caused by evolution of protons (H^+^) and hydroxide (OH^−^) ions at the electrodes diffuse from the electrodes outward^5^,^27^. Fundamental studies on tissue ablation by electrolysis have shown that pH changes are indicative of electrolytic tissue ablation^19^. It was found that pH dyes marked gels^27^,^28^,^29^, could be used to study, monitor and image electrolysis.

Our work draws from a 1957 paper by Meiboom, Luz and Gill who studied proton relaxation times in water as a function of pH^30^. During the last several decades, Magnetic Resonance Imaging (MRI) was used to study pH changes in many bio-medical settings with various methods and for various applications. For example, the effect of intracellular pH, as well as blood and tissue oxygen tension on T1 relaxation in the rat brain were studied in Ref. 31. Measurements of pH changes due to ischemia in the brain, in relation to amine and amide protons were reported in Refs 32, 33. Measurements of pH changes due to kidney failure with an MRI-CEST pH responsive contrast agent, Iopamidol were presented in Ref. 34. An evaluation of a range of MRI-active pH indicators for food applications is found in Refs 35, 36. In Ref. 37, it was shown that calf muscle T2 changes correlate with pH, PCr recovery and oxidative phosphorylation. Most relevant to this study is the work of Schilling et al, who have found that changes in intracellular pH, affect the relaxation time of T2 in brain tissue^38^. While an abundance of papers exist on the use of MRI for detecting changes in pH, no publication on the use of MRI for monitoring the development of pH fronts during electrolysis was found by our literature search. The primary goal of this study is to explore the hypothesis that pH fronts, produced by electrolysis, can be detected with MRI, for possible application in monitoring and controlling cell ablation with electrolysis. As briefly illustrated by the above literature citations, there are many methods that could be used for detecting pH changes in tissue with MRI. Inspired by references^30^,^38^, we chose to explore our hypothesis with basic T1 weighted and T2 weighted based sequences for water.

To explore the hypothesis we conduct an experimental study using a pH dye stained physiological saline agar-gel based phantom as a model for a living tissue, from an electrochemical standpoint. The pH dye generates a continuous temporal and spatial image of the effected region, but is restricted to use in transparent systems. In addition we have collected discrete measurements using a pH probe – with the limitations of these measurements in mind. In the study we compare images obtained with MRI to optical images acquired using pH-sensitive dyes and the discrete pH data. The MRI imaging sequences used were T1 weighted (T1W), T2 weighted (T2W) and Proton Density (PD). The optical images were acquired using pH-sensitive dyes embedded in the agar phantom that is exposed to electrolysis. In addition to validating the MRI-based approach using pH-sensitive dyes, we demonstrate a biological application of our MRI work by comparing a spatial map of bacterial viability exposed to electrolysis with the MRI image of the phantom during electrolytic treatment. Our results are promising, and invite further experimental explorations.

## Results

### MRI Experiment Results

**Figure 1:**
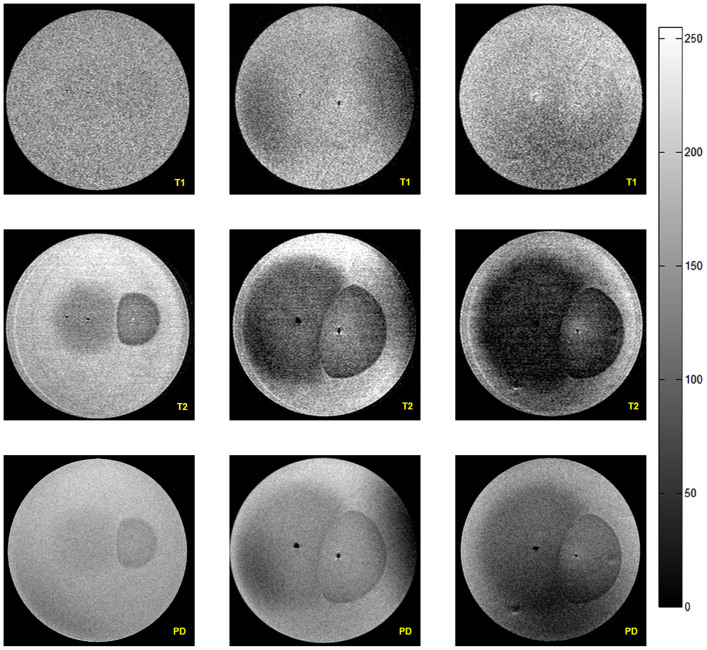
Comparative MRI imaging results. Each row corresponds to a sequence modality (Top to bottom: T1, T2, PD). Each column corresponds to a stimulation voltage (Left to right: 3 V, 6 V, 9 V).

**Figure 2:**
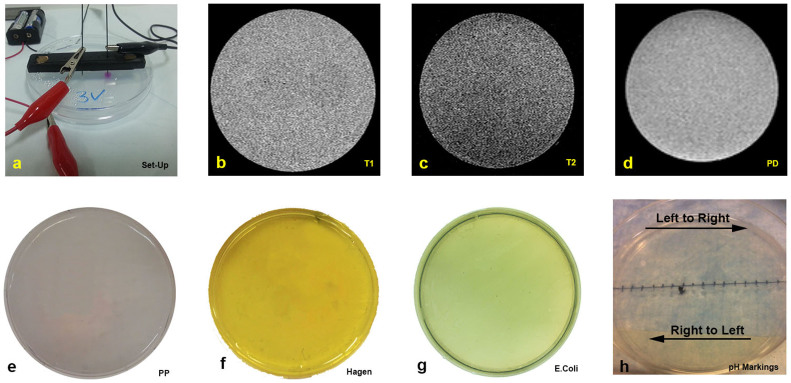
Experimental setup and control images for different experimental modalities used in this work. (a) Experimental setup, (b) T1 control study, (c) T2 control study, (d) PD control study (e) Phenolphtalein 1%, (f) Hagen wide range pH indicator dye, (g) E. Coli control study.

As described in our methods, the agar plates were scanned before the administration of electrolytic treatment with the following sequences: T1W, T2W and PD. Then the gel plates were electrolytically treated for 15 minutes using three different voltages: 3 V, 6 V, and 9 V. After administering the treatment, the plates were immediately positioned in a pediatric head coil, and inserted into the MR scanner. MR sequences with the same pre-treatment parameters were then acquired. The MRI parameters are presented in Table 1. To facilitate comparison of results, figure 1 brings together images obtained for the three voltages and the three MRI sequences. The three columns are for the voltages of 3 V, 6 V and 9 V, from left to right, respectively. The rows from top to bottom are for the following sequences: T1W, T2W, and PD, respectively. All the images are for a standard Petri dish with the same diameter, 8.5 cm. The electrolysis was administered via the same device, positioned at the same place for all the experiments, as constrained by the application rig in figure 2a. The position of the electrodes can be seen in some images as the two black traces (void of signal) at the centerline of the Petri dish. The distance between the electrodes was 2 cm. In all the images the anode is on the left and the cathode is on the right.

The first row of figure 1 shows images taken with the T1W sequence. The signal from the treated volume is isointense to hypointense. It is isointense for the lower voltage of 3 V and becomes slightly hypointense with the increasing applied voltage. The pH change front is barely distinguishable for the 9 V treatment. The second row shows results obtained with the T2W sequence. The margin of the electrolysis-affected region is marked with dotted yellow line. Hypointense signal can be seen in the treated region, with the signal intensity decreasing with increasing voltage. The affected region near the anode is larger than near the cathode. The altered pH front appears diffused in the anode-affected region and well-delineated in the cathode affected region. The interface between the cathode affected region and the anode affected region is distinct and visible. It is also note-worthy that in the cathode-affected region, the intensity adjacent to the cathode decreases with increasing voltage. The images produced with PD sequences, presented in the third row, show a generally similar pattern to that described for the T2W sequence produced images. Images produced with the PD sequence show a hypo intense signal with lower intensity relative to the T2W sequence produced images.

### pH Dye Experiment

**Figure 3:**
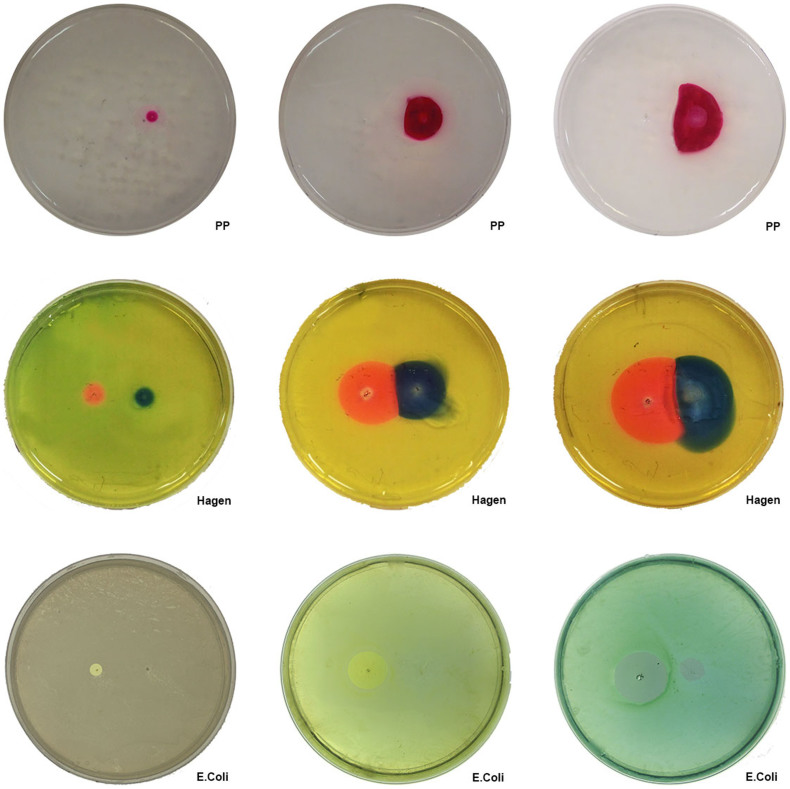
Comparative pH dyes and bacterial viability results. Each row corresponds to a control modality (Top to bottom: Phenolphtalein 1% pH indicator,Hagen pH indicator, E. Coli bacterial viability). Each column corresponds to a stimulation voltage (Left to right: 3 V, 6 V, 9 V).

For the pH dye experiments we have infused the agar gel phantom described in our methods with two pH sensitive indicator dyes. Figure 3 shows results obtained from the pH dye experiments. To facilitate the comparison of results, Figure 3 brings together images obtained for the three voltages and results from the two-pH dyes infused gels. The three columns are for voltages of 3 V, 6 V and 9 V, from left to right, respectively. The first row shows results obtained with phenolphthalein staining. The phenolphthalein stain produces a distinct pink color in the pH range from 8.2 to 12. The first row shows, as expected, an impression in only the cathode region on the right. The margins of the marked regions indicate a minimal pH of 8.2. For a voltage of 3 V, the change of pH front takes a circular shape, most likely of a pH of 8.2. Increasing the voltage increases the size of the change in pH-affected area. Similar to the MRI images, the cathode-affected front collides with the anode produced front at a line between the electrode and cathode. The outer margin of the lesion that has a circular shape is most likely at a pH of 8.2, while the central line could be at any pH in the range of pH 8.2 to pH 12. It is interesting to note that immediately near the electrode for the 9 V voltage the intensity of the image is reduced compared to a region further away from the electrode.

The second row shows the results of pH staining using the Hagen wide range pH testing kit. The cathodic region on the left is marked with a distinct blue color which indicates a basic pH in the vicinity of 8.3, while the anodic region on the right is marked with pink color which corresponds to pH level of 6.4. We have used the color-matching card provided by the manufacturer to establish the pH ranges. For 3 V, the pH change affected regions have a circular shape. Increasing the voltage increases the size of the affected region. Just as for the other pH dye, and MRI images, the larger voltages lead to colliding pH fronts, which can observed as a straight line. Several interesting phenomena can be seen in the Hagen stained samples. First, for voltages of 6 V and 9 V, the areas immediately adjacent to the electrodes appear discolored relative to the surrounding stained areas. Furthermore, on the cathode side at 6 V and 9 V there is a drop of fluid, which was observed on the top of the gel. For 9 V, some of the dye has leaked into this drop and stained it.

### pH probe experiments

**Figure 4:**
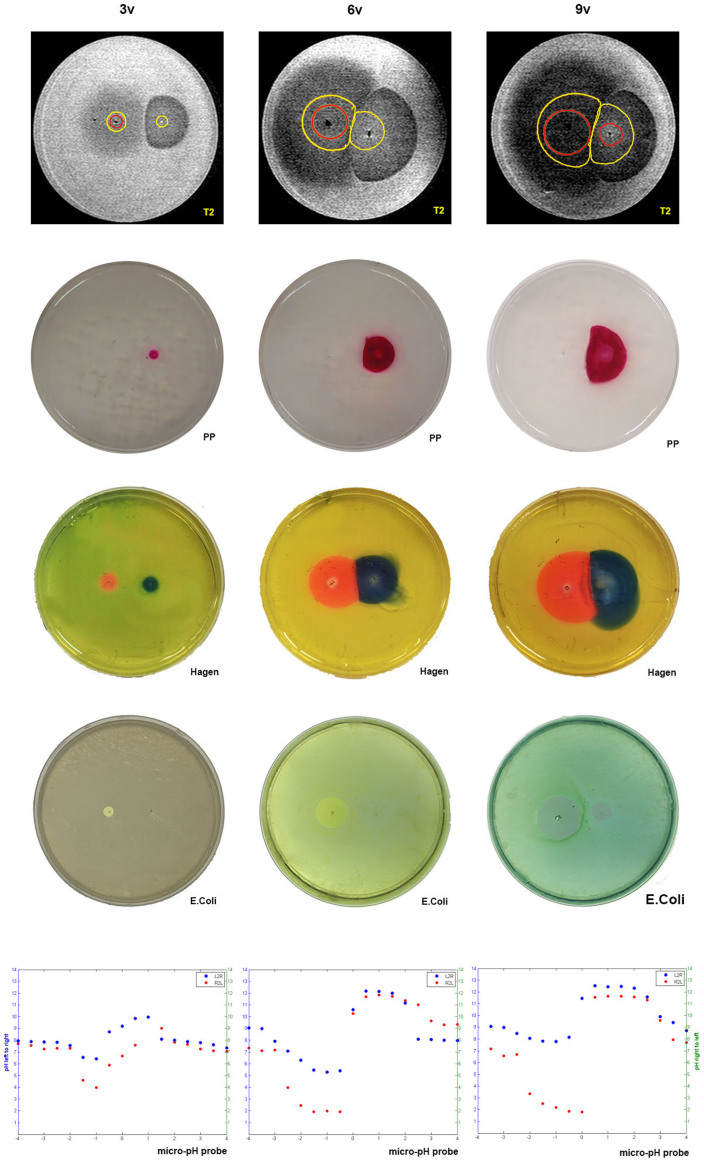
Comparative pH dyes and bacterial viability results. Each row corresponds to a control modality (Top to bottom: Phenolphtalein 1% pH indicator,Hagen pH indicator, E. Coli bacterial viability, Local pH measurements taken by pH probe). Each column corresponds to a stimulation voltage (Left to right: 3 V, 6 V, 9 V).

To correlate the pH data captured by MRI and the pH sensitive dyes, the pH measurements made by the pH probe are presented in Table 2 and visualized graphically in the fifth row of Figure 4. The discrete pH measurements are presented on a single chart for each voltage. The effect of growing voltage is clearly seen: the extreme value of pH reached on both fronts is more deviant from the baseline normal pH. The data is presented in two forms, with the measurements made from left to right and from right to left. The precision of the pH reading is 0.1 and the precision of the data point location is +/−0.5 mm. The difference between the left to right measurement curve and the right to left measurement curve is indicative of the systematic error of this measurement.

### Bacterial Viability Experiment

To demonstrate the relevance of our work to a biological model, electrolytic stimulation was applied to an agar dish plated with *E. Coli* bacteria. The third row in figure 3 shows optical images of a bacterial viability pattern after treatment with 3 V, 6 V and 9 V for 15 minutes, captured using a digital camera after 24 hour growth period. The anodic region on the right is marked with a clear bactericidal region increasing in area with increasing voltage. The cathodic region on the left is significantly smaller in terms of bactericidal area and is barely observable in the 3 V image.

## Discussion

As mentioned in the introduction, our hypothesis that MRI can be used to image the pH fronts during electrolysis is based on reports in refs 30 and 38. As can be observed in the T1W images (Figure 1 first row), the treated volume exhibits hypointense to isointense signal, which indicates that the effect of electrolysis is minimal on T1W signal. A T1-weighted sequence produces an image where the signal contrast is determined by the differences in T1 relaxation times. The tissue signal in a T1 weighted imaging mode is inversely proportional to its T1 relaxation time. A short echo time (TE) is used to minimize T2-weighting together with a short repetition time (TR). A T1-weighted image is typically characterized by dark fluid signal due to the long T1 relaxation time of water. This result is consistent with the original findings of Meiboom, Luz and Gill who studied proton relaxation times in water as a function of pH^30^ and show that T1 in water does not change in the range of from pH 2 to pH 12,

Visible changes are produced by electrolysis in T2 weighted images (Figure 1, second row). In the T2-weighted imaging mode, the signal contrast is determined by differences in T2 relaxation times. The tissue signal in a T2 weighted image is proportional to its T2 relaxation time. A long repetition time (TR) is used to minimize T1-weighting together with a long echo time. The results in figure 1 are also consistent with the original findings of Meiboom, Luz and Gill^30^. Their findings show that T2 in water is strongly affected by changes in pH and it increases symmetrically around pH 7 with an increase and decrease in pH. Shilling et al.^38^ provide a mechanistic explanation for their observed changes in T2 with changes in pH in the brain which is consistent with the findings of Meiboom *et. al.* by noting that “The effect of pH on spin-spin relaxation time (T2) might be explained by the fact that at pH 7.0, i.e., in the neutral environment, water molecules build larger hydrogen-bound mediated clusters than in the acid or base ranges. The reduced mobility leads to a prolonged correlation time for the dipolar interactions, which leads to a shortening of T2”^39^. Figure 1 shows that the electrolysis affected region near the anode is larger than that near the cathode. This difference makes physical sense and can be attributed to the relative radii of protons (*H*^+^, 0.88 *fm*) and hydroxide ions (*OH*^−^, 110 *pm*). Due to their relative smaller size, the protons are more mobile hence contributing to a larger extent to the conductivity increase around the anode. The increased mobility causes the pH region around the anode to be larger than around the cathode.

Proton Density (PD) is defined as the number of proton spins per unit volume of a tissue. Proton density may differ from the true water content due to short T2 components, which are not seen in MRI. So PD-weighted imaging where the T1 and T2 effects are minimized leads to images whose contrast is determined primarily by the spin (proton) density. This requires a short TE and long TR. In figure 1, the third row shows the process of electrolysis generated PD MRI images, which correspond well with the T2W images. This confirms that the observed images are related to the electrolysis caused diffusion of protons and hydroxide ions.

In figure 3, the first and the second rows show the results of the process of electrolysis obtained with pH stained dyes. While the optical pH results cannot be quantitatively compared with the MR images, because the pH dyes have a restricted range, both the MRI and pH dyes images show similar phenomena and trends. The observed affected zone increase in both modalities with an increase in voltage, which is consistent with the increased production of electrolytic compounds with voltage. The anode and cathode electrolysis affected regions meet at the same location in both the pH dye images and the MRI images. The pH dye results, in particular the second row of figure 3, show some additional interesting physical phenomena. The effect relates to the observed drops of water on the surface of the gel, during electrolysis with 6 V and 9 V. It is known that in an electric field, water moves by electro-osmosis from the anode to the cathode^6^. Therefore, during electrolysis, the gel near the anode tends to dehydrate while water accumulates near the cathode. This is the source of the water observed in the second row of figure 3. We tentatively suggest that this electro-osmotic migration of water is responsible for the discoloration adjacent to the cathode observed with both MRI and pH dyes.

In figure 3, row three shows viability results from electrolysis treated *E. Coli*, grown on the surface of the gel. This part of the work is clinically relevant because electrolysis is becoming an important method for sterilizing surfaces and wounds, considering the growing antibiotic resistances of microorganisms^40^. The pattern of cell ablation observed here is consistent with electrolytic ablation and further supports the idea that the MRI detected changes are relevant to electrolysis. Figure 3, row three shows that the extent of cell ablation increases with the voltage and charge delivered as expected from a pH ablation process driven by electrolysis. It is also well established that the electrolytic products of the anode are more effective at cell ablation than the products of the cathode^6^. This is also confirmed in this study, which shows a much larger ablation zone near the anode than near the cathode.

In Figure 4, a comparison between MRI images, pH dye based images, discrete pH measurements and bacterial viability data shows that all the experiments, produce qualitatively similar results with respect to the effect of voltage on the affected area and with respect to the difference between the anodic and cathodic regions. A quantitative comparison is not possible because the pH dyes and the bacterial viability images represent limited ranges of pH, while the discrete pH measurements suffer from systematic errors. However, the results from the different imaging techniques show that increasing the voltage (charge delivered) increases the affected area in both the anode and cathode affected volume, the anodic front advances faster than the cathodic front, and the anode and cathode affected regions meet on the line perpendicular to the line connecting the electrodes. Local pH measurements presented in the fifth row of Figure 4 are consistent qualitatively with the other means of pH monitoring, as they indicate more extreme pH ranges for higher voltages.

Figure 4 was brought here to summarize the results. The first row shows the T2W MR image onto which we have superimposed the outline of the pH dye image (rows two and three) and the outline of the viability experiment (row four). It is interesting to notice that the interface between the anode and the cathode affected zones lie on the same line in the MRI image and the pH dye image – suggesting that they both represent the same phenomenon. The overall shape of the pH dye image is similar to the MRI image. The affected zone observed with MRI is larger than that observed with dyes, because the range of changes that can be observed with MRI is not restricted by a certain pH dye value. The extent of cell ablation is substantially less than the extent of the region in which MRI detects changes in pH. In the past, studies on the effect of electrolysis on cell death were carried out using pH probes or pH dyes. This study suggests that MRI could become a useful tool in fundamental research on the effect of electrolysis on cells.

To address the temporal resolution of the MRI based approach and its relevance in the context of a typical length of an electrolysis procedure, we'd like to note that the resolution is limited by the time it takes to run a single scan which is given by *N*·*N_p_*·*T_r_* where *N* represents the number of signals averaged (NSA excitations), *N_p_* represents the size of the encoding matrix and *T_r_* represents the time to repetition. When multiple echoes are acquired after each excitation, this allows for accelerating the scan time and occasionally is referred to as the Turbo Spin Echo (TSE) or Turbo Factor. In such a case, the time given above should by divided by the Turbo Factor. For a typical choice of parameters, e.g. in our case study, the scan time falls in the range of 2–4 minutes. This is shorter than a typical length of an electrolytic treatment procedure and is much shorter than the time it would take to generate a complete scan with discrete pH measurements. This further suggests that MRI could be a potential method to study electrolysis in tissue.

## Methods

### Experimental Outline

The experiment was designed to allow for a comparison between different images of pH fronts produced by the electrolysis of a physiological saline solution phantom. The images were generated by various MRI sequences and compared with: a) optical images acquired using pH-sensitive dyes embedded in a physiological saline agar solution phantom treated with electrolysis and b) bacterial *E. Coli* model, grown on a phantom and treated by applying the same electrolysis protocol. Each experimental study was done separately. In addition we have also collected a set of discrete measurements using a micro pH probe.

### Tissue model

Our tissue model consists of a physiological saline based agar gel phantom with electrical conductivity designed to simulate that of a tissue. To construct the phantom, 1% Bacto-Agar (Fisher Scientific) was mixed with 0.9 g/l Sodium Chloride (Fisher Scientific) in distilled water. The solution was then brought to a boil and poured into 85 mm diameter Petri dishes. The same dish dimension was used in all the studies. The conductivity of the agar phantom was measured to be approximately 0.14 *S*/*m* which is close to the range of hepatic tumor conductivity^41^.

### Experimental procedure

The experimental setup is shown in Figure 2.a. We have used two disposable graphite electrodes made of pencil lead (Pentel super HB 0.7 mm), to avoid contamination with products of electrolysis from the electrodes. The electrodes, mounted in a horizontal holder were placed perpendicularly to the gel phantom in the Petri dish. The electrodes were inserted 7 mm deep into the gel at a distance of 2 cm between their centers. The electrodes are connected to constant voltage batteries. We used 3 V, 6 V and 9 V batteries. The electrolysis process lasted 15 minutes. While typical electrolysis stimulation is administered using a fixed current source, we have used a fixed voltage source and have taken current measurements during the procedure (data not shown) for charge dosage estimation purposes. The overall delivered charge dosages over the 15 minutes stimulation period were approximately 0.9 C, 1.8 C and 2.9 C, for 3 V, 6 V and 9 V, respectively. These charge dosages fall within the range of a typical electrolytic ablation therapy stimulation^3^,^42^. Identical experiments were done separately for MRI imaging, pH dyes based optical imaging, discrete pH measurements and the E. Coli viability model.

### MRI Experimental Model

The phantom models were scanned, before and after electrolysis in a clinical 1.5 T MRI system (Philips Achieva SE) using a SENSE pediatric coil. The specific MRI parameters of each sequence are listed in Table 1. The mean acquisition time for each sequence was 3 minutes. Images shown in the paper were taken in the following order: T1W, T2W and PD. For later comparison with the MR images produced after electrolysis, figures 2.b, 2.c and 2.d show the baseline images for the sequences introduced above, respectively. In repeated experiments (results not shown) we changed the order and the time after the end of electrolysis at which the various sequences were taken and found no measurable effect of the time at which the images were acquired on the dimensions of the affected region.

### pH-Sensitive Dye Model

As a control study, we used 2 distinct, pH sensitive dyes in order to estimate the boundary of the region where the pH has changed due to electrolysis. The dyes employed were 1) Phenolphtalein 1% by Sigma (turns pink in pH range of 8.2–12) and 2) Nutrafin pH wide range by Hagen (indicates pH by color in the range of 4.5–9). The dye manufacturer instructions prescribed the concentration of the last dye. We used a digital camera (Casio Exilim EX-ZR100) to acquire optical images of the experimental chamber before and after electrolysis and correlate these images with the images acquired using MRI and bacterial viability. For later comparison with images produced after electrolysis, figures 2.e and 2.f, show the baseline images prior to electrolysis for the two dyes used, respectively.

### Discrete measurements with micro pH probes

To produce numerical values for pH we have used a MI-4146 Micro-combination pH probe (Microelectrodes, Inc. 40 Harvey Rd., Bedford, NH) with a response time of 5 seconds to 15 seconds in a stirred solution and a pH meter pH150 Oakton (625 East Bunker Court., Vernon Hills, IL). The experimental procedure is similar to that used in the MRI study and the pH sensitive dye study. We have marked a line through the diameter of the Petri dish and marked off every 5 mm (see Fig 1.h). The two electrodes were placed at a distance of 2 cm between them. Similarly to the previous experiments we applied voltages of 3 V, 6 V and 9 V for 15 minutes. After the electro-stimulation, the electrodes were removed, and pH measurements were made, starting with the marking furthest away from the dish center. To minimize and evaluate the effect of diffusion on the systematic errors of this method, in one set of the experiments the reading was performed from left to right, and in another set of experiments the reading was performed from right to left. The pH probe was placed over the mark and into the gel, making certain that the tip was covered. We waited until the reading was stabilized, usually between 15 and 25 seconds. The reading was recorded and the pH probe flushed and moved over to the next mark until the entire dish was transversed. One transverse reading took about 20 to 25 minutes. (No new electrolytic products were produced during the measurement period, however the pH changed during the measurements because of diffusion). While the actual data recorded is with a precision of 0.1, the accuracy in relation to the colorimetric dye and MRI measurements is low. Therefore, the discrete pH measurement should be viewed as a rough indication of the actual values. We also considered measuring pH during the electro-stimulation process, however, we found, in other studies, that the insertion of an active pH probe during electrolysis distorts the reading because of the effect of the current on the pH probe.

### Bacterial Model

Lyophilized *E. Coli* of HB101 strain (BioRad) were grown in LB broth overnight and plated on LB broth based agar gel filled petri dishes. The LB broth for the overnight growth consisted of 1% BactoTryptone (BD), 0.5% Yeast Extract (BD), 1% NaCl (Sigma Aldrich) and 1.5% Agarose (Sigma Aldrich). 6 mm glass beads (Sigma Aldrich) were used for plating to ensure uniform coverage. After plating, the beads were removed and the plates were incubated for 15 minutes at 37°. The conductivity of the gel was measured around 0.2 *S*/*m*. After electrolysis the Petri dishes were covered and incubated for 24 hours. We used a digital camera (Casio Exilim EX-ZR100) to acquire optical images of the areas where bacterial growth was inhibited and correlated these images with the images acquired using MRI and gels with pH dyes. For later comparison with images produced after electrolysis, figure 2.g shows the baseline images for an untreated with electrolysis, cell growth plate.

## Conclusion

In summary, we report experimental findings that support the hypothesis that electrolysis induced pH-changes can be detected with MRI. Our results indicate the feasibility of using MRI as a means to monitor dynamic changes in local pH level of a biological sample during an electrolysis process. Our work uses an agar-based gel model with conductivity in the range of a biological tissue, and is validated vs. optical images utilizing pH indicator dyes. In addition we demonstrate the relevance of our work in the biological context by correlating bacterial viability data with MRI measurements. It may be interesting to work on developing different MRI techniques for detecting pH, using MRI markers. Reference^30^, suggests that it may be also possible to develop MRI sequences that detect discretely various ranges of pH, because T2 seems to be very sensitive to pH. Our results are promising, and invite further experimental explorations on the use of MRI for studying electrolysis and in clinical practice.

## Author Contributions

A.M. has performed the bacterial and ph-dye experiments, prepared figures 3–4 and wrote the paper. M.H. has performed the MRI experiments, prepared table 1, figures 1–2 and wrote the paper. L.R. prepared table 2 and performed the micro-pH experiments. B.R. has conceived the experiments and contributed to writing the paper.

## Figures and Tables

**Table 1:** MRI parameters used in the study

Sequence	Coil	TR [ms]	TE [ms]	FOV [mm]	Slice thickness	Num. slices	# of excitations NSA	Recon. Matrix size
T1W TSE	SENSE-Pediatric Head coil	500	17	120	2 mm	4	2	512
T2W TSE	SENSE-PED-HEAD	1000	100	120	2 mm	4	2	512
PD	SENSE-PED-HEAD	5000	30	120	2 mm	4	2	512

**Table 2:** pH measurements taken by the micro pH probe. Each voltage contains RL and LR columns: measurements taken from right to left and left to right respectively

Distance from edge[mm]	3 V RL	3 V LR	6 V RL	6 V LR	9 V RL	9 VLR
0	7.94	7.7	9.05	7.35	9.08	7.15
0.5	7.89	7.55	8.98	7.09	8.98	6.57
1	7.86	7.25	7.92	7.15	8.46	6.7
1.5	7.83	7.32	7.06	3.98	8.06	3.35
2	7.55	7.3	6.3	2.47	7.82	2.53
2.5	6.55	4.6	5.47	1.92	7.8	2.19
3	6.43	3.97	5.29	1.98	8.15	1.87
3.5	8.72	5.87	5.4	1.93	11.45	1.81
4	9.19	6.67	10.6	10.25	12.52	11.53
4.5	9.84	7.59	12.17	11.7	12.45	11.63
5	9.95	9.96	12.15	11.84	12.46	11.63
5.5	8.10	9.02	11.98	11.73	12.31	11.57
6	8.01	7.81	11.15	11.36	11.58	11.29
6.5	7.88	7.63	8.1	11.02	9.91	9.57
7	7.8	7.26	8.07	9.65	9.41	7.95
7.5	7.6	7.1	8.00	9.3	8.7	7.71
8	7.35	7.04	7.97	9.34	n/a	n/a

## References

[b1] Amory R. A treatise on electrolysis and its therapeutical and surgical treatement in disease. (William Woof & Co., 1886).

[b2] Nordenstrom B. E. W. Preliminary clinical trials of electrophoretic ionization in the treatement of malignant tumors. IRCS Medical Sc. 6, 537 (1978).

[b3] Nordenstrom B. E. Electrochemical treatment of cancer. I: Variable response to anodic and cathodic fields. Am J Clin Oncol 12, 530–536 (1989). 2556014

[b4] Nilsson E., Berendson J. & Fontes E. Development of a dosage method for electrochemical treatment of tumours: a simplified mathematical model. Bioelectroch Bioener 47, 11–18, 10.1016/s0302-4598(98)00157-3 (1998).

[b5] Nilsson E., Berendson J. & Fontes E. Electrochemical treatment of tumours: a simplified mathematical model. J Electroanal Chem 460, 88–99, 10.1016/s0022-0728(98)00352-0 (1999).

[b6] Nilsson E. *et al.* Electrochemical treatment of tumours. Bioelectrochemistry 51, 1–11, 10.1016/s0302-4598(99)00073-2 (2000). 10790774

[b7] von Euler H., Nilsson E., Olsson J. M. & Lagerstedt A. S. Electrochemical treatment (EchT) effects in rat mammary and liver tissue. In vivo optimizing of a dose-planning model for EChT of tumours. Bioelectrochemistry 54, 117–124, 10.1016/s1567-5394(01)00118-9 (2001). 11694391

[b8] von Euler H., Nilsson E., Lagerstedt A. S. & Olsson J. M. Development of a dose-planning method for electrochemical treatment of tumors: A study of mammary tissue in healthy female CD rats. Electro Magnetobiol 18, 93–+ 10.3109/15368379909012903 (1999).

[b9] Bergues Pupo A. E., Bory Reyes J., Bergues Cabrales L. E. & Bergues Cabrales J. M. Analytical and numerical solutions of the potential and electric field generated by different electrode arrays in a tumor tissue under electrotherapy. Biomed Eng Online 10 10.1186/1475-925x-10-85 (2011). PMC3247137 21943385

[b10] Placeres Jimenez R. *et al.* 3D Stationary Electric Current Density in a Spherical Tumor Treated With Low Direct Current: An Analytical Solution. Bioelectromagnetics 32, 120–130, 10.1002/bem.20611 (2011). 21225889

[b11] Turjanski P. *et al.* pH front tracking in the electrochemical treatment (EChT) of tumors: Experiments and simulations. Electrochimica Acta 54, 6199–6206, 10.1016/j.electacta.2009.05.062 (2009).

[b12] Camue Ciria H. M. *et al.* Antitumor effects of electrochemical treatment. Chn J Cancer Res 25, 223–234, 10.3978/j.issn.1000-9604.2013.03.03 (2013). PMC3626978 23592904

[b13] Yoon D.-S. *et al.* Introduction of electrochemical therapy (EChT) and application of EChT to the breast tumor. J. Breast Canc 10, 162–168 (2007).

[b14] Czymek R. *et al.* Electrochemical Treatment: An Investigation of Dose-Response Relationships Using an Isolated Liver Perfusion Model. Saudi J Gastro 17, 335–342, 10.4103/1319-3767.84491 (2011). PMC3178922 21912061

[b15] Griffin D., Dodd N., Zhao S., Pullan B. & Moore J. V. Low-level direct electrical current therapy for hepatic metastases. I. Preclinical studies on normal liver. Brit J Cancer 72, 31–34 (1995). 7599063 10.1038/bjc.1995.272 PMC2034160

[b16] Griffin D., Dodd N. J., Moore J. V., Pullan B. & Taylor T. The effects of low-level direct current therapy on a preclinical mammary carcinoma: tumour regression and systemic biochemical sequelae. Brit J Cancer 69, 875–878 (1994). 8180017 10.1038/bjc.1994.169 PMC1968917

[b17] Miklavčič D. *et al.* Tumor treatment by direct electric current-tumor temperature and pH, electrode material and configuration. Bioelectrochem Bioenerget 30, 209–220 (1993).

[b18] Miklavčič D., Fajgelj A. & Serša G. Tumor treatment by direct electric current: electrode material deposition. Bioelectrochem Bioenerget 35, 93–97 (1994).

[b19] Finch J. G. *et al.* Liver electrolysis: pH can reliably monitor the extent of hepatic ablation in pigs. Clin Sci 102, 389–395 (2002). 11914100

[b20] Olaiz N., Suarez C., Risk M., Molina F. & Marshall G. Tracking protein electrodenaturation fronts in the electrochemical treatment of tumors. Electrochem Commun 12, 94–97, 10.1016/j.elecom.2009.10.044 (2010).

[b21] Ivic M. L. A., Perovic S. D., Zivkovic P. M., Nikolic N. D. & Popov K. I. An electrochemical illustration of the mathematical modelling of chlorine impact and acidification in electrochemical tumour treatment and its application on an agar-agar gel system. J Electroanal Chem 549, 129–135, 10.1016/s0022-0728(03)00251-1 (2003).

[b22] Moore E. W. Determination of pH by the glass electrode: pH meter calibration for gastric analysis. Gastroenterology 54, 501–507 (1968). 5652504

[b23] Takmakov P. *et al.* Characterization of local pH changes in brain using fast-scan cyclic voltammetry with carbon microelectrodes. Anal Chem 82, 9892–9900 (2010). 21047096 10.1021/ac102399n PMC2995839

[b24] Venton B. J., Michael D. J. & Wightman R. M. Correlation of local changes in extracellular oxygen and pH that accompany dopaminergic terminal activity in the rat caudate–putamen. J Neurochem 84, 373–381 (2003). 12558999 10.1046/j.1471-4159.2003.01527.x

[b25] De Beer D., Schramm A., Santegoeds C. M. & Kuhl M. A nitrite microsensor for profiling environmental biofilms. Appl Environ Microbiol 63, 973–977 (1997). 16535560 10.1128/aem.63.3.973-977.1997 PMC1389125

[b26] Chu K. F. & Dupuy D. E. Thermal ablation of tumours: biological mechanisms and avdances in therapy. Nat Rev Cancer 14, 199–208 (2014). 24561446 10.1038/nrc3672

[b27] Turjanski P. *et al.* pH front tracking in the electrochemical treatment (EChT) of tumors: Experiments and simulations. Electrochimica Acta 54, 6199–6206 (2009).

[b28] Turjanski P. *et al.* The role of pH fronts in reversible electroporation. PLoS One 6, e17303 (2011). 21559079 10.1371/journal.pone.0017303 PMC3084685

[b29] Maglietti F. *et al.* The Role of Ph Fronts in Tissue Electroporation Based Treatments. PLoS One 8, e80167 (2013). 24278257 10.1371/journal.pone.0080167 PMC3836965

[b30] Meiboom S., Luz Z. & Gill D. Proton Relaxation *In Water*. J Chem Phys 27, 1411–1412, 10.1063/1.1744015 (1957).

[b31] Kettunen M. I., Grohn O. H. J., Silvennoinen M. J., Penttonen M. & Kauppinen R. A. Effects of intracellular pH, blood, and tissue oxygen tension on T-1 rho relaxation in rat brain. Magn Reson Med 48, 470–477, 10.1002/mrm.10233 (2002). 12210911

[b32] McVicar N. *et al.* Quantitative tissue pH measurement during cerebral ischemia using amine and amide concentration-independent detection (AACID) with MRI. J Cerebr Blood F Met 34, 690–698, 10.1038/jcbfm.2014.12 (2014). PMC3982091 24496171

[b33] Zong X., Wang P., Kim S. G. & Jin T. Sensitivity and Source of Amine-Proton Exchange and Amide-Proton Transfer Magnetic Resonance Imaging in Cerebral Ischemia. Magn Reson Med 71, 118–132 (2014). 23401310 10.1002/mrm.24639 PMC3655131

[b34] Longo D. L., Busato A., Lanzardo S., Antico F. & Aime S. Imaging the pH evolution of an acute kidney injury model by means of iopamidol, a MRI-CEST pH-responsive contrast agent. Magn Reson Med 70, 859–864, 10.1002/mrm.24513 (2013). 23059893

[b35] Evans S. & Hall L. Evaluation of a range of MRI-active pH indicators using a multiple-sample method. Aiche Journal 51, 1541–1547, 10.1002/aic.10387 (2005).

[b36] Evans S. & Hall L. Measurement of pH in food systems by magnetic resonance imaging. Can J Chem Eng 83, 73–77 (2005).

[b37] Schmid A. I. *et al.* Exercising calf muscle T-2 changes correlate with pH, PCr recovery and maximum oxidative phosphorylation. Nmr Biomed 27, 553–560, 10.1002/nbm.3092 (2014). 24610788 PMC4260669

[b38] Schilling A. M., Blankenburg F. B., Bernarding J., Heidenreich J. O. & Wolf K. J. Intracerebral pH affects the T2 relaxation time of brain tissue. Neuroradiology 44, 968–972, 10.1007/s00234-002-0873-0 (2002). 12483440

[b39] Schilling A., Blankenburg F., Bernarding J., Heidenreich J. & Wolf K. Intracerebral pH affects the T2 relaxation time of brain tissue. Neuroradiology 44, 968–972 (2002). 12483440 10.1007/s00234-002-0873-0

[b40] Piggott J. M., Berney H., Hurley E. & Clair J. Planar flexible electrode for use in wound sterilization. Paper presented at *24th Annual International Conference of the Engineering in Medicine and Biology Society*. Annual Fall Meeting of the Biomedical Engineering Society, 1929–1930 vol. 1923, 10.1109/iembs.2002.1053098 (2002).

[b41] Laufer S., Ivorra A., Reuter V. E., Rubinsky B. & Solomon S. B. Electrical impedance characterization of normal and cancerous human hepatic tissue. Physiol Meas 31, 995 (2010). 20577035 10.1088/0967-3334/31/7/009

[b42] Nordenström B. Biologically closed electric circuits: Clinical, experimental and theoretical evidence for an additional circulatory system. (Ursus Medical AB, 1983).

